# Interstitial Lung Disease With Respiratory Failure After COVID-19 mRNA Vaccination

**DOI:** 10.7759/cureus.58491

**Published:** 2024-04-17

**Authors:** Soichi Maruyama, Taro Takahashi, Daisuke Kobayashi, Yoneko Hayase, Yukihiko Sugiyama

**Affiliations:** 1 Department of Respiratory Medicine, Nerima Hikarigaoka Hospital, Tokyo, JPN; 2 Department of Pathology, Nerima Hikarigaoka Hospital, Tokyo, JPN

**Keywords:** long-term oxygen therapy, bronchoscopy, steroid, covid-19 mrna vaccine, drug-induced interstitial lung disease (di-ild)

## Abstract

A septuagenarian woman developed dyspnea on the day following a fifth vaccination. Just before vaccination, a chest X-ray showed no abnormalities, but after the fifth vaccination, bilateral diffuse ground-glass opacities were detected. Bronchoalveolar lavage revealed a lymphocyte predominance and transbronchial lung biopsy revealed growth of the alveolar epithelium, along with organized polypoid granulation tissues in the alveolar ducts and bronchioles. Despite the administration of corticosteroids, imaging revealed persistent fibrosis, and she required long-term oxygen therapy. Although recent reports indicated that corticosteroids are effective for drug-induced interstitial lung disease related to COVID-19 mRNA vaccination, this case presented a somewhat different clinical manifestation.

## Introduction

One of the COVID-19 mRNA vaccines, BNT162b2 (Pfizer-BioNTech), exhibits remarkable efficacy, boasting a 95% success rate in preventing disease exacerbation [1]. Common adverse effects associated with these vaccines include mild symptoms such as fever, fatigue, and headache [2]. However, severe adverse events such as anaphylaxis and thrombosis, though rare, have been reported [3,4]. Notably, drug-induced interstitial lung disease (DI-ILD) following vaccination is exceedingly uncommon. Despite this rarity, we encountered a case where dyspnea manifested immediately post-BNT162b2 vaccination, with subsequent imaging revealing interstitial lung disease. Histopathological examination confirmed DI-ILD. This case underscores the importance of vigilance within the medical community.

## Case presentation

A septuagenarian woman was admitted to our hospital complaining of dyspnea on exertion. In December 2022, a chest radiograph taken as part of a medical check-up revealed no abnormalities in the lung fields. Ten days later, the patient received a fifth dose of BNT162b2. Subsequently, she began to experience dyspnea the following day, and her condition deteriorated throughout the month before her first hospital visit.

**Figure 1:**
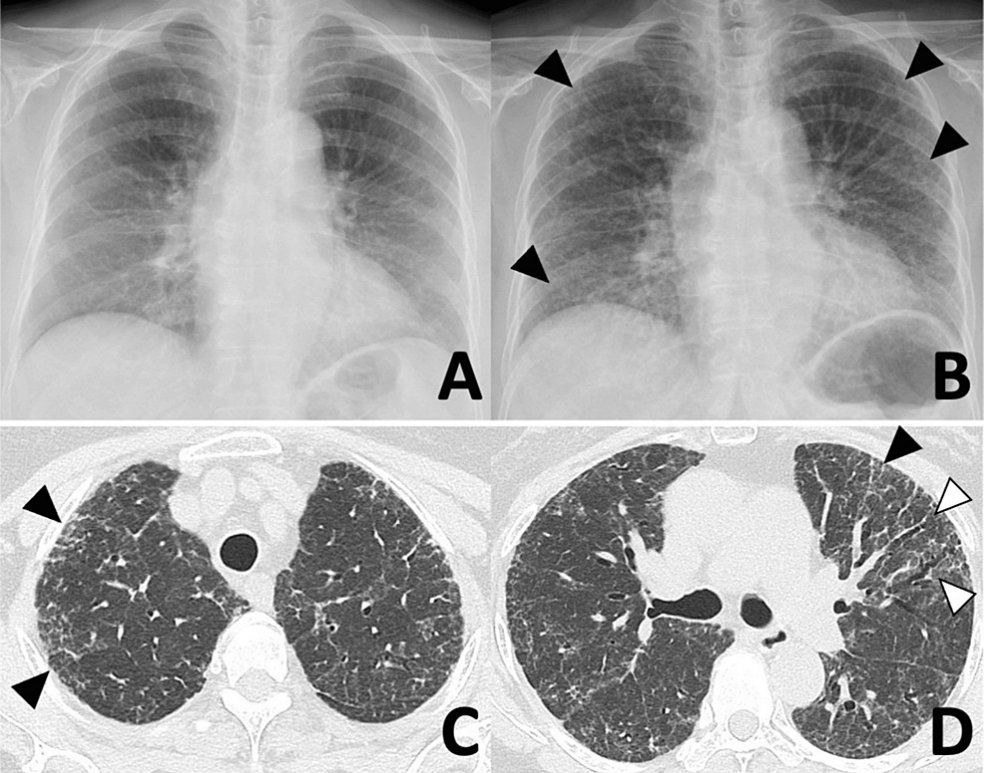
Chest radiograph and high-resolution CT of the chest Before the fifth BNT162b2 mRNA vaccination, a chest radiograph showed no abnormalities (A). After vaccination, infiltrative shadows (black arrows) were detected in the lower field of both lungs (B). Chest computed tomography revealed bilateral ground-glass opacities (black arrows), thickened interlobular septal walls, and traction bronchiectasis (white arrows) (C, D).

The patient had no history of exposure to dust inhalation, birds, mold, or humidifiers. Furthermore, she had no previous history of smoking or COVID-19 infection. She had been receiving fenofibrate therapy for several years. On admission, she required nasal cannula oxygenation at 4 L/min during physical exertion. Auscultation revealed fine crackles, audible predominantly in the lower thoracic region. No rashes or arthralgia were noted. Blood tests revealed an elevation of Krebs von den Lungen-6 (KL-6) and surfactant protein-D (SP-D) (Table 1). Antibodies associated with connective tissue disease and the *Trichosporon asahii* antibody were not detected. Urine *Streptococcus pneumoniae* antigen, urine *Legionella pneumophila* antigen, and sputum cultures were all negative. Two PCR samples (one from the nasopharynx and the other from saliva) were negative for acute respiratory syndrome coronavirus-2 (SARS-CoV-2). A chest radiograph taken after the fifth BNT162b2 revealed infiltrative shadows on the lower field of both lungs (Figures 1A, 1B). Chest computed tomography (CT) revealed bilateral ground-glass opacities (GGOs), with thickened interlobular septal walls and traction bronchiectasis (Figures 1C, 1D).

**Table 1:** Blood laboratory tests WBC: White blood cell; AST: Aspartate aminotransferase; ALT: Alanine aminotransferase; LD: Lactase dehydrogenase; ALP: Alkaline phosphatase; BUN: Blood urea nitrogen; KL-6: Krebs von den Lungen-6; SP-D: Surfactant protein-D; CCP: Cyclic citrullinated peptide; ab: antibody; dsDNA: Double-stranded deoxyribonucleic acid; Sm: Smith; SS-A: Ro; SS-B: La; U1-RNP: U1-ribonucleoprotein; Scl-70: Topoisomerase I; ARS: Aminoacyl tRNA synthetase; MDA5: Melanoma differentiation-associated gene 5; MPO: Myeloperoxidase; ANCA: Antineutrophil cytoplasmic antibody; PR3: Proteinase 3

		Range			Range
Complete blood cell count			Immunoserological examination		
WBC, /μL	6260	3300-8600	C-reactive protein, mg/dL	0.19	<0.14
Neutrophil count, %	70.7	-	antinuclear ab, times		
Lymphocyte count, %	14.7	-	KL-6, IU/mL	4191	105.3-401.2
Monocyte count, %	6.5	-	SP-D, ng/mL	1008	<110
Eosinophile count, %	7.5	-	Rheumatoid factor, IU/mL	5	5.0-15.0
Basophile count, %	0.6	-	Anti CCP ab	(-)	(-)
Haemoglobin, g/dL	13.2	11.6-14.8	Anti dsDNA ab	(-)	(-)
Platelet, /μL	37.4×10^4^	15.8-34.8	Anti Sm ab	(-)	(-)
Biochemical examination			Anti SS-A ab	(-)	(-)
Total bilirubin, mg/dL	0.6	0.4-1.5	Anti SS-B ab	(-)	(-)
AST, IU/L	28	13-30	Anti U1-RNP ab	(-)	(-)
ALT, IU/L	17	7-23	Anti Scl-70 ab	(-)	(-)
LD, IU/L	399	124-222	Anti ARS ab	(-)	(-)
ALP, IU/L	150	104-338	Anti Jo-1 ab	(-)	(-)
Albumin, g/dL	4.5	4.1-5.1	Anti MDA5 ab	(-)	(-)
BUN, mg/dL	10.3	8.0-20.0	MPO-ANCA	(-)	(-)
Creatinine, mg/dL	0.57	0.46-0.79	PR3-ANCA	(-)	(-)
Sodium, mEq/L	141	138-145	*Trichosporon asahii* ab	(-)	(-)
Potassium, mEq/L	4.2	3.6-4.8			
Chlorine, mEq/L	107	101-108			

**Figure 2:**
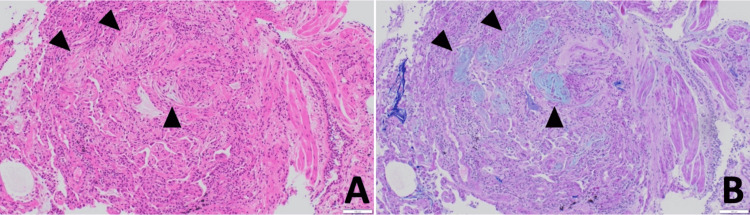
Histopathological findings obtained by transbronchial lung biopsy Strong fibrous growth from the alveolar wall into the alveoli (black arrows), reactive type 2 alveolar epithelial growth, and bronchial epithelialization were observed. (A) Hematoxylin-Eosin stain: ×100. (B) Periodic Acid-Schiff-Alcian Blue staining: ×100.

Bronchoscopy was performed, and bronchoalveolar lavage (BAL) fluid from the right B5 showed lymphocyte and eosinophil proliferation (55% and 10%, respectively) and an increased CD4/CD8 ratio (6.6); bacterial cultures were negative. The transbronchial lung biopsy (TBLB) of the right B8a revealed strong fibrous growth from the alveolar wall into the alveoli, along with reactive type II alveolar epithelial growth and bronchial epithelialization. These findings were consistent with organizing pneumonia with fibrosis (Figure 2).

Based on the patient's clinical history, radiological features, laboratory data, and histopathological findings, a diagnosis of DI-ILD caused by the BNT162b2 mRNA vaccination was made. After bronchoscopy, the patient received 500 mg of methylprednisolone for three days, followed by prednisolone (PSL) at 30 mg/day. Following treatment initiation, the patient's symptoms subsided, and levels of both KL-6 and SP-D decreased. The patient was discharged from the hospital on Day 23; however, a follow-up CT scan seven weeks after her first visit showed persistent bilateral GGOs with fibrosis and traction bronchiectasis; therefore, long-term oxygen therapy was continued.

## Discussion

Here, we diagnosed COVID-19 mRNA vaccine-related DI-ILD in a patient, showing no other cause of the sudden-onset ILD other than a recent COVID-19 mRNA vaccination.

BNT162b2 mRNA is encapsulated within lipid nanoparticles. When the mRNA reaches the target cell, it is taken up, and the encoded SARS-CoV-2 spike protein is expressed. The spike protein is then presented on the cell surface, where it is detected by dendritic cells, which in turn trigger cellular and humoral immune responses [5]. However, it is suggested that fragments of antigens and/or associated peptides may be released into the circulation and that the lipid nanoparticles may have proinflammatory effects [6]. Such immune responses may be associated with DI-ILD. According to a survey by the Japanese Ministry of Health, Labour, and Welfare (as of March 12, 2023), an estimated 294,416,519 doses of BNT162b2 have been administered. The total number of cases of interstitial pneumonia was 81 (0.000028%); therefore, sequelae are extremely rare. However, we do not know how many of these cases were DI-ILD.

We searched for recent reports of COVID-19 mRNA vaccine-related DI-ILD [7-14]. The characteristics of these patients include the appearance of respiratory symptoms soon after vaccination, onset of disease during the first or second vaccination period, high levels of KL-6 and SP-D (range 214-4084 U/mL and 73.1-675.5 ng/mL, respectively), an organizing pneumonia pattern on chest CT, a high percentage of lymphocytes in BAL fluid, and high responsiveness to steroids. In the current case, interstitial pneumonia appeared only after the fifth vaccination, and the response to steroids was poor. The latter was likely because the patient visited our outpatient clinic one month after the onset of the disease. In addition, lung fibrosis likely progressed during this time, causing irreversible changes to the lung structures.

Among the known case reports, TBLB was performed in two cases [12,14]. In all of these cases, polypoidal organization and fibrosis were observed in the alveolar space, and the fibrosis was relatively uniform at early time points. The pathological picture was different from that of cryptogenic organizing pneumonia due to strong inflammatory cell infiltration coupled with atypical alveolar epithelial cells. DI-ILD caused by the BNT162b2 mRNA vaccine is suspected to be a manifestation of organizing pneumonia with severe inflammation.

## Conclusions

COVID-19 mRNA vaccine-related DI-ILD is extremely rare, and there is insufficient information to prove a causal relationship. It is important to note that the benefits of the COVID-19 vaccination outweigh the risks, as the vaccines are highly effective at preventing severe disease and hospitalization. We highly recommend that individuals get vaccinated; however, it should be noted that DI-ILD can occur after COVID-19 mRNA vaccination, as in this case. Further studies on COVID-19 mRNA vaccine-related DI-ILD are required.
